# Nonlinear optical induced lattice in atomic configurations

**DOI:** 10.1038/s41598-020-67540-2

**Published:** 2020-08-07

**Authors:** Sijia Hui, Feng Wen, Xiaojun Yu, Zhiping Dai, Irfan Ahmed, Yunpeng Su, Yanpeng Zhang, Hongxing Wang

**Affiliations:** 1 Key Laboratory for Physical Electronics and Devices of the Ministry of Education, School of Science, Shaanxi Key Lab of Information Photonic Technique, Institute of Wide Bandgap Semiconductors, Xi’an Jiaotong University, Xi’an, 710049 China; 2 School Automation, Northwestern Polytechnical University, Xi’an, 710072 China; 3 College of Physics and Electronic Engineering, Hengyang Normal University, Hengyang, 421002 China; 4 Department of Electrical Engineering, Sukkur IBA University, Sukkur, 65200 Pakistan; 5 State-Owned Sida Machinery Manufacturing, Xianyang, 712201 China

**Keywords:** Mathematics and computing, Nonlinear optics, Mathematics and computing, Nonlinear optics, Mathematics and computing

## Abstract

Traditional artificial lattice with untunable refractive index have been restricted to flexible applied to kinds of micro medium imaging. This study proposes a novel approach to quantifying lattice using nonlinear optically induced periodic lattice, which possesses a striking feature of tunable refractive index, to further broaden current knowledge of optical imaging equipment. We conduct self-dressed and dual-dressed nonlinear four-wave mixing (FWM) signal modulation in the atoms by using the dressing effect of standing waves, and then investigate the space amplitude modulation and synthetization (amplitude and phase) modulation of the electromagnetic induced lattice (EIL) of FWM signal at the atom surface. The EIL presented in the far-field diffraction region confirms that diffraction intensity of the FWM signal can be easily transformed from zero-order to higher-order based on the dispersion effects. The tunable EIL with ultra-fast diffraction energy change can contribute to a better understanding of nonlinear process and provides a further step toward developing two-dimensional nonlinear atomic higher-resolution.

## Introduction

Optical imaging, such as grating structure imaging, is an useful method to investigate the microscopic substance and it can effectively modify the coherence of incident lights to obtain a changeable or adaptable optical image. However, the traditional grating structures may lead to phase mismatches between the grating period and the Bragg’s period, which inevitably causes severe wavelength shifts and then degenerates the grating properties. In a perspective way, the major problem with this kind of artificial grating is the constant refractive index original from the untunable medium interval energy levels. Nevertheless, the issue of tunable gratings proposed by Ling et. al. ^1^ has received considerable critical attention, which utilize standing waves to produce a spatially periodic structure defined as electromagnetically induced grating (EIG) ^2^. Therefore, the optical controlled EIG becomes a key instrument in many applications, such as all-optical switching ^3^, light storage ^4^, optical bi-stability^5^, wave-shaping a bi-photon spectrum^6^, and beam splitting and fanning ^7^. Recently, the developments in the field of EIG have led to a renewed interest in vertical dimension. It has been reported that a two-dimensional optically induced atomic lattice (EIL) is realized experimentally to observation of diffraction pattern^8^. It is now well established the one-dimensional and two-dimensional optical tunable EIL, however, the influence of EIL on nonlinear process has remained unclear.

In this paper, we offer a new model to investigate the modulation of EIL^9,10^based on nonlinear process by introducing electromagnetically induced transparency (EIT) ^11^ and then examine the transformed types of EIL in the far-field diffraction regime. The research question in this study focused on the combination of nonlinear four-wave mixing (FWM) process and standing waves approaches. In practice, the all-optically controlled specialty in our scheme originating from the tunable energy level, which is defined as lattice states, is realized by using the dressing effect of two-dimensional standing waves. The lattice states can suppress or enhance the FWM outputs ^12,13^ in both x and y- directions simultaneously by exploiting weak light nonlinearity EIT effect. As a result, the two-dimensional amplitude and synthesized EILs of periodic adjustable FWM signal are presented through choosing different parameters at atom surface. In far-field diffraction, the synthesized EIL will diffract to be a phase EIL with diffraction energy transferring from zero-order to first-order, while the amplitude EIL can only diffract to finite first-order diffraction term with mostly zero-order diffraction term remained. This study with the weak light nonlinearity induced as an useful method to investigate the interactions of optical field with atom medium, which could be applied in many potential applications, such as the nondestructive and lens-less imaging of ultra-cold atoms and molecules, the weak signal detection, and so on.

The advantages of our scheme are as following. Firstly, the EIL is not only reconfigurable by choosing different optical parameters, but also can avoid many experimental errors in the cumbersome process, so as to maintain the experimental accuracy. Secondly, the multi-beam interference can effectively change the atom medium energy interval derived by dressing effect and realizes some complicated lattice structures, such as the quasi-crystals, defect mediated lattice, honeycomb lattice, Bessel lattice, virtual lattice, etc. Thirdly, the phase shift that corresponds to the dispersion effect of nonlinear process can switch the types of EIL rapidly as well as redistribute the diffraction energy from zero-order to higher-order significantly. Meanwhile, the all-optical controlled weak light nonlinearity processes could be applied in many potential aspects without damaging the medium, such as all-optical switching ^14–17^, routing^18,19^, light storage^20^, material optical properties probing^21^ and beam splitting and fanning^22^, etc.

## Basic theory

**Figure 1:**
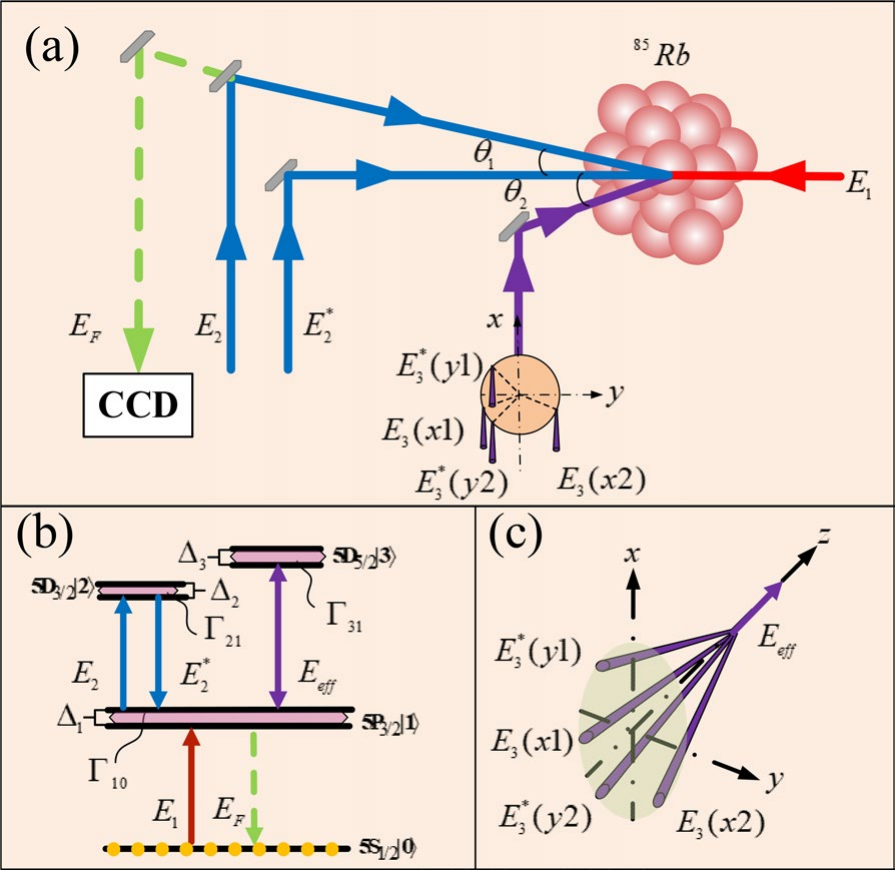
Configuration of the weak light nonlinear signal generate in the four energy levels structure which driven by two-dimensional standing waves $E_{eff}$ (**a**) The setup of the light path to realize the FWM signal. (**b**) FWM mode generates in the diagram of Y energy level diagram of ^85^Rb atom with dressed field $E_{eff}$. (**c**) The diagram to form two-dimensional standing waves field by multi-beam interference including $E_{3} (x1)$ and $E_{3} (x2)$, which interfere with each other in x-direction, as well as $E_{3}^{*} (y1)$ and $E_{3}^{*} (y2)$ propagation in y-direction.

**Figure 2:**
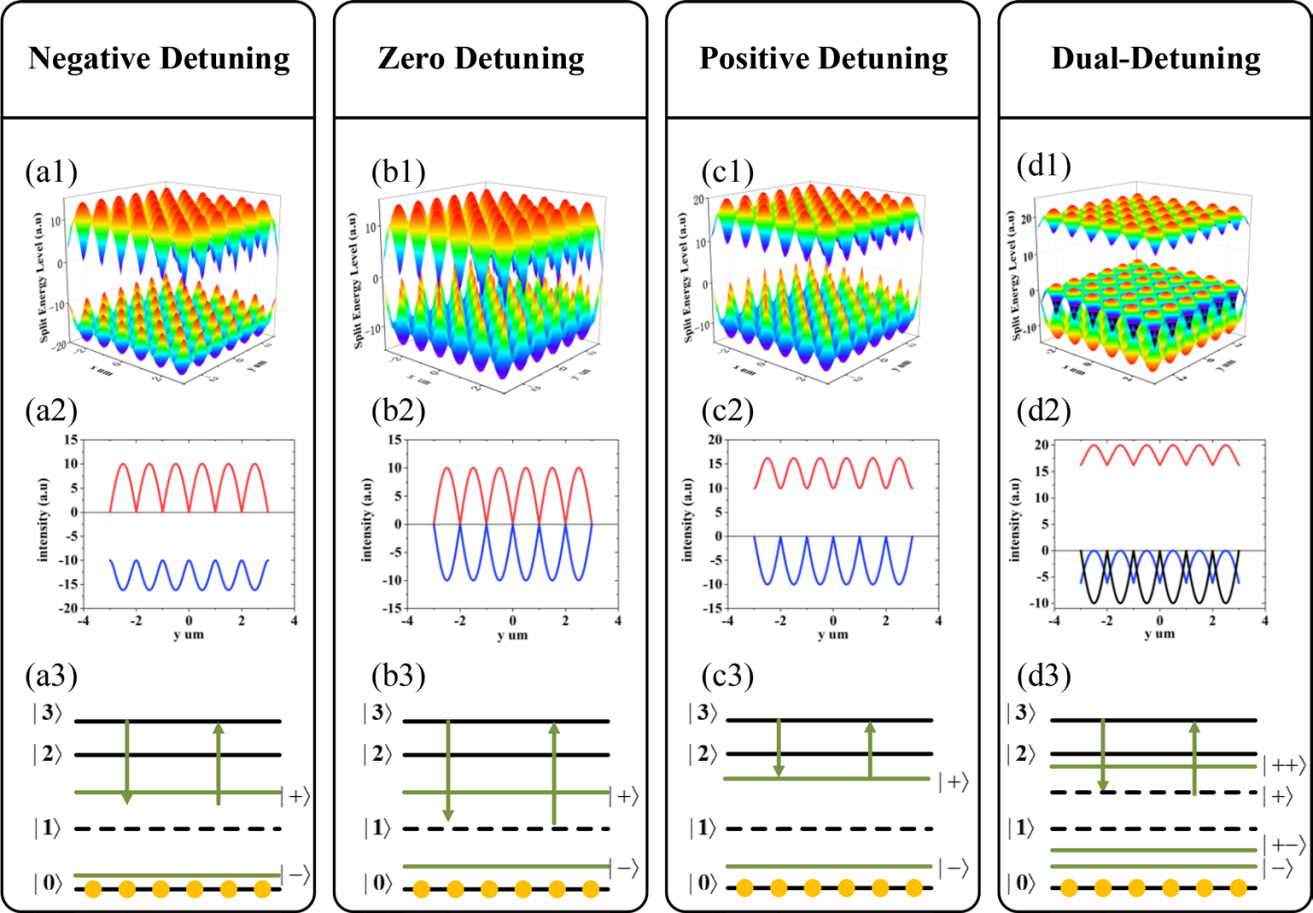
Standing wave $G_{3} (x,y)$ with detuning $\Delta_{2}$, $\Delta_{3}$ periodically modulate the dressed energy level distribution. (**a1**) Three-dimensional energy level splitting with single dressed effect at $\Delta_{3} { = } - {10}MHz$. (**a2**) Splitting energy level distribute along the y-direction at x = 0 μm. (**a3**) The split energy diagram with additional field dressed effect. The energy levels $| + \rangle$ and $| - \rangle$ are dressed energy states. (**b1**), (**b2**), (**b3**) corresponding to (**a1**), (**a2**), (**a3**) at $\Delta_{3} { = 0}MHz$. (**c1**), (**c2**), (**c3**) corresponding to (**a1**), (**a2**), (**a3**) at $\Delta_{3} { = 10}MHz$. (**d1**), (**d2**), (**d3**) corresponding to (**a1**), (**a2**), (**a3**) at $\Delta_{{2}} { = 10}MHz$, $\Delta_{3} { = 0}MHz$.

Firstly, we demonstrate the way to realize the lattice states which could change the intensity output of the weak light nonlinear FWM signal ($E_{F}$). The process generated in an ensemble of ultra-cold ^85^Rb atoms is shown in Fig. 1(a). The Y energy level model consists of four levels: a ground state ${|0}\rangle$ ($5S_{1/2}$) and three excited states $|1\rangle$ ($5P_{3/2}$), ${|2}\rangle$ ($5D_{3/2}$) and ${|3}\rangle$ ($5D_{5/2}$) as illustrated in diagram of Fig. 1(b). Considering the generating process of FWM signal, the transition between states ${|0}\rangle \to {|1}\rangle$ is simulated by the probe field $E_{{1}}$ with frequency $\omega_{{1}}$ and detuning $\Delta_{{1}} = \omega_{{1}} { - }\omega_{{{10}}}$. The level ${|1}\rangle$ is coupled to level ${|2}\rangle$ by pump fields $E_{2}$ and $E_{2}^{*}$ with a slight propagating angle $\theta_{{1}}$. The frequency of $E_{2}$ and $E_{2}^{*}$ is $\omega_{{2}}$ and detuning $\Delta_{{2}} { = }\omega_{{2}} { - }\omega_{{{21}}}$. It should be noted that the pump and probe beams propagate in the opposite direction satisfying the two-photon Doppler-free condition. The process of FWM signal generation shown in the configuration of Fig. 1(b) that the signal propagates opposite to $E_{2}$^23^ and satisfies the momentum conservation $k_{F} = k_{1} + k_{2} - k_{2}^{*}$^24–27^. Secondly, we demonstrate the formation mechanism of lattice states by using standing waves. The upper level $|3\rangle$ is coupled to level $|{1}\rangle$ by the standing waves propagating along the x- and y-directions. As show in Fig. 1(c), $E_{3} (x1)$ and $E_{3} (x2)$ ($E_{3}^{*} (y1)$ and $E_{3}^{*} (y2)$) with a slight angle $\theta$ form a standing wave along the x-direction (y-direction) and then a two-dimensional standing waves field $E_{eff}$ is produced in x–y plane. $E_{eff}$ incident in atoms with a tiny angle $\theta_{2}$ to propagation direction of $E_{2}^{*}$, but counter-propagate to $E_{1}$ in order to eliminate the Doppler effect. Therefore, this kind of procedure is not only suitable for the ultra-cold ensemble, but also could be applied to the thermal atomic systems ^28^. The expression of standing wave intensity is $\left| {G_{{3}} (x,y)} \right|^{2} = \left| {\Omega_{c} \sin (\pi x/a)} \right|^{2} + \left| {\Omega_{c} \sin (\pi y/a)} \right|^{2}$, where $\Omega_{c} = \mu_{i} E_{i} /\hbar (i = 1,2,3...)$ is Rabi frequency of standing waves with a and b are the periods along x and y-directions, respectively. As a result, the periodically changed energy level defined as lattice states are introduced by the two-dimensional standing wave $G_{{3}} {\text{(x,y)}}$ shown in Fig. 2.

Next, we concentrate on the way to modulate the energy interval. Level $|1\rangle$ is splitted into dressed states $| + \rangle$ and $| - \rangle$ when standing waves couple with the energy levels transition $|1\rangle \to |3\rangle$ and eigenvalues of splitting states $| \pm \rangle$ are $\lambda_{ \pm } = \Delta_{3} /2 \pm \sqrt {\Delta_{3}^{2} /4 + \left| {G_{3} (x,y)} \right|^{2} }$^29^. Obviously, the periodic characteristic of two-dimensional lattice states attributes to standing wave $G_{{3}} (x,y)$, while $\Delta_{{3}}$ and $G_{{3}} (x,y)$ both decided the splitting distance between lattice states $| \pm \rangle$. For instance, setting $\Delta_{{3}} = - 10MHz$, the splitting states $| \pm \rangle$ shown in Fig. 2(a1) are not symmetric with level $|1\rangle$, which determined by eigenvalue $\lambda_{ - } = \Delta_{3} /2 - \sqrt {\Delta_{3}^{2} /4 + \left| {G_{3} (x,y)} \right|^{2} }$, and the sinusoidal periodical characteristic of splitting states is emphasized in Fig. 2(a2) along y-direction (x = 0 μm) as well as an asymmetric dressed states diagram illustrated in Fig. 2(a3). When setting $\Delta_{{3}} = 0MHz$, the dressed states $| \pm \rangle$ are split symmetrically as shown in Fig. 2(b1), which correspond to resonance transition $|1\rangle \to |3\rangle$. The periodic changed state $| - \rangle$ is much higher than the state when $\Delta_{3} = - 10MHz$ in Fig. 2(b2) leading to the reduced energy level distance in Fig. 2(b3) compared to Fig. 2(a3). Likewise, the varied lattice sates show the same asymmetric distribution both in x–y plane in Fig. 2(c1) at $\Delta_{3} = 10MHz$, as well as the periodic property shown in Fig. 2(c2) and the adjustable energy interval diagram in Fig. 2(c3). Then we take the self-dressed effect of fields $E_{2}$ and $E_{2}^{*}$ into consideration and the eigenvalues of splitting states are transformed into $\lambda_{ \pm \pm } = (\Delta_{2} + \lambda_{ \pm } )/2 \pm \sqrt {(\Delta_{2} - \lambda_{ \pm } )^{2} /4 + \left| {G_{2} } \right|^{2} }$. When $E_{{2}}$ dressed on the splitting states $| \pm \rangle$ following the standing wave resonantly coupled to levels $|{1}\rangle \to |3\rangle$, the dual-dressed states $| + \pm \rangle$ are generated. Figure 2(d1) demonstrates the two-dimensional periodic dual-dressed lattice states along x and y-axis distributed asymmetrically and simplified for secondary splitting energy level diagram in Fig. 2(d3). The dominant characteristic of splitting energy level is that having periodic nodes and antinodes displayed in Fig. 2(d2). At nodes, only the dressed function of $E_{{2}}$ and $E_{2}^{*}$ results single energy level split, whereas at antinodes, standing wave $G_{3} (x,y)$ can also affect the dressed energy and bring the secondary energy level splitting. As a result, the intensity of weak light nonlinear FWM signal ($E_{F}$) is periodically changed at different positions of nodes and antinodes. Furthermore, taking the third order nonlinear process with nonlinear coefficient $\chi^{(3)}$ into consideration, we obtained the Liouville pathways as follows: $\rho_{{{00}}}^{(0)} \mathop{\longrightarrow}\limits{{\omega_{1} }} \mathop{\longrightarrow} \rho_{{G_{2} + G_{3} (x,y) \pm 0}}^{(1)} \mathop{\longrightarrow}\limits{{\omega_{2} }} \mathop{\longrightarrow} \rho_{{{20}}}^{(2)} \mathop{\longrightarrow}\limits{{\omega_{2} }} \mathop{\longrightarrow} \rho_{{G_{2} + G_{3} (x,y) \pm 0}}^{(3)}$. The weak light nonlinearity process of the dressed density matrix of the produced $E_{F}$ signal can be expressed as follows:

1 $$ \rho_{{G_{2} + G_{3} (x,y) \pm 0}}^{(3)} = \frac{{G_{a} }}{{d_{2} (d_{1} + \left| {G_{2} } \right|^{2} /d_{2} + \left| {G_{3} (x,y)} \right|^{2} /d_{3} )^{2} }} $$

where $$G_{a} = - iG_{1} G_{2} (G_{2}^{^{\prime}} )^{*} \exp (ik_{F} \cdot r)$$, $$G_{1}$$, $$G_{2}$$, and $$G_{2}^{*}$$ are the Rabi frequencies of $$E_{1}$$, $$E_{2}$$, $$E_{2}^{*}$$, respectively. And $$d_{1} = \Gamma_{10} + i\Delta_{1}$$$$d_{2} = \Gamma_{20} + i(\Delta_{1} + \Delta_{2} )$$$$d_{3} = \Gamma_{30} + i(\Delta_{1} + \Delta_{3} )$$. $$\Gamma_{ij}$$ is the decay rate between the energy level $$|i\rangle$$ and $$|j\rangle$$$$i,j = 0,1,2,3$$ signal. More detail can be found in Supplementary. The $$E_{F}$$ generated from $$\rho_{{G_{2} + G_{3} (x,y) \pm 0}}^{(3)}$$ is changed periodically due to standing waves can modulate the nonlinear coefficient. In other words, the weak light nonlinear $$E_{F}$$ signal can be modulated by periodical lattice states.

## Results and discussion

**Figure 3:**
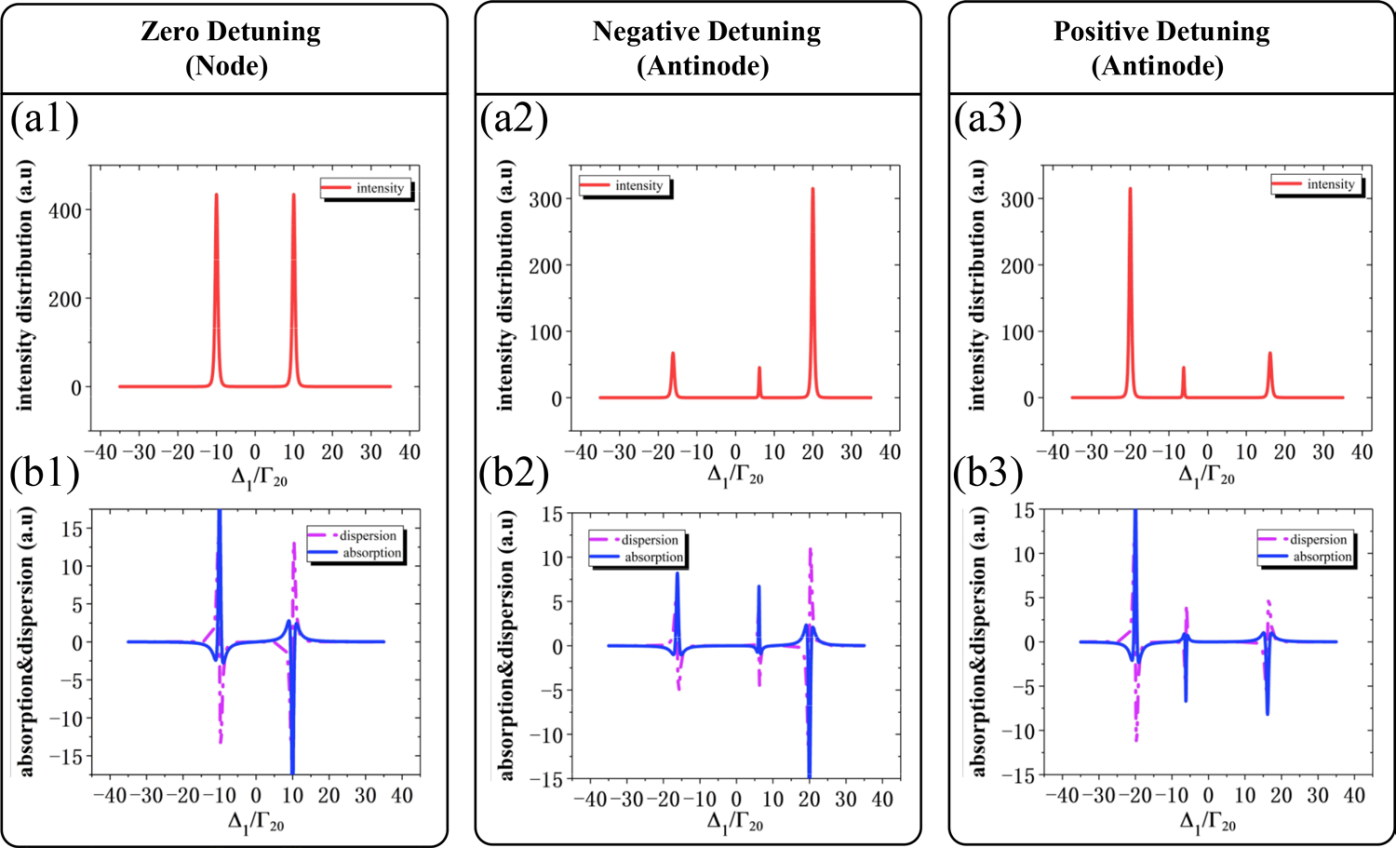
The FWM signal intensity distribution with single dressed and dual-dressed effects. (**a1**) The intensity distribution at the node of x = 0, y = 1 with $\Delta_{2} = 0MHz$ shows two intensity peaks attribute to single dressed effect. (**a2**) The intensity distribution at the antinode of x = 0.5, y = 0.5 with $\Delta_{2} = - 10MHz$ shows three intensity peaks due to dual-dressed effects. (**a3**) The intensity distribution at the antinode of x = 0.5, y = 0.5 with $\Delta_{2} = 10MHz$ obtains symmetrical intensity peaks with (**a2**). (**b1**), (**b2**), (**b3**) The absorption curve and dispersion curve corresponding to (**a1**), (**a2**), (**a3**), respectively. The other parameters are $\Omega_{{1}} = {3}MHz$, $\Omega_{{2}} = {\text{10M}}Hz$, $\Gamma_{10} { = 1}M{\text{Hz}}$, $\Gamma_{20} = {0}{\text{.2}}MHz$, $\Gamma_{30} = {0}{\text{.2}}MHz$.

In this section, we will not only investigate the simulation results to study how the $$E_{F}$$ intensity distribution of signal affected by the single and double dressed lattice states, but also comparing the variation of $$E_{F}$$ signal intensity at node (x = 0,y = 1) and antinode (x = 0.5,y = 0.5) by scanning $$\Delta_{1}$$ at different values of $$\Delta_{{2}}$$. Firstly, the two intensity peaks distributed at the node (x = 0, y = 1) is apparent shown in Fig. 3(a1), which means there is only single self-dressed effect on level $$|1\rangle$$ and split it into dressed states $$| \pm \rangle$$ with eigenvalues $$\lambda_{ \pm } = \Delta_{2} /2 \pm \sqrt {\Delta_{2}^{2} /4 + \left| {G_{2} } \right|^{2} }$$. According to the dressed theory, when $$\Delta_{1}$$ satisfies the condition $$\Delta_{1} + \lambda_{ \pm } = 0$$, the intensity distribution of the $$E_{F}$$ signal appears with dual peaks shown in Fig. 3(a1). Likewise, we scan $$\Delta_{{1}}$$ at the antinode (x = 0.5, y = 0.5), corresponding to consider the standing wave dressed effect, the dressed states $${|} \pm \rangle$$ will further split into secondary dressed states $$| + \pm \rangle$$, $${|} - \rangle$$ ($$| - \pm \rangle$$, $$| + \rangle$$) and the eigenvalues of states $$|{ + } \pm \rangle$$ are $$\lambda_{{{ + } \pm }} { = }\Delta_{{2}} {/2 + }\Delta_{{3}} {/4 + }\frac{{1}}{{2}}\lambda_{ + } \pm \sqrt {\left( {\Delta_{{2}} { - }\Delta_{{3}} {/2 - }\lambda_{ + } } \right)^{{2}} /4{ + }\left| {G_{2} } \right|^{2} }$$.Unlike the single dressed effect in Fig. 3(a1), the $$E_{F}$$ signal intensity enhance by satisfying the condition $$\Delta_{1} + \lambda_{ + } + \lambda_{ + \pm } = 0$$ and $$\Delta_{1} + \lambda_{ - } = 0$$, corresponding to three peaks in Fig. 3(a2) and (a3) at $$\Delta_{3} = - 10,10MHz$$, respectively. The explanation mechanisms for states $$| + \pm \rangle$$$${|} - \rangle$$ are as the same for the states $$| - \pm \rangle$$, $$| + \rangle$$, so we focus on explaining the states $$| + \pm \rangle$$$${|} - \rangle$$. Besides, when $$\Delta_{{1}}$$ satisfies the condition $$\Delta_{1} + \Delta_{3} = 0$$, $$E_{F}$$ signal intensity will be decreased in comparison with intensity enhancement condition. Therefore, we define the enhancement condition as $$\Delta_{1} + \lambda_{ + } = 0$$ ($$\Delta_{1} + \lambda_{ + } + \lambda_{ + \pm } = 0$$) and suppression condition as $$\Delta_{1} + \Delta_{3} = 0$$. Secondly, we separate $$\chi^{(3)}$$ into a real part $$\chi^{^{\prime}}$$ and imagine part $$\chi^{^{\prime\prime}}$$ to further study the essential effect of enhancement and suppression on the $$E_{F}$$ signal. The dispersion and amplitude curve at node (x = 0, y = 1) as shown in Fig. 3(b1) from $$\Delta_{1} = - 10MHz$$ to $$\Delta_{1} = 10MHz$$, we observe that both the $$\chi ^{\prime}$$ and $$\chi ^{\prime\prime}$$ zero value of an EIT window generation and then the atom ensembles become a transparent medium for the probe field $$E_{1}$$, leading to no FWM signal production. While the $$E_{F}$$ signal peaks are realized at the cross points of amplitude curve peak and sharp shift dispersion curve. At the antinode (x = 0.5, y = 0.5), the zero-values region both in real part and imaginary part is split into double zero-values region because standing wave field ($$E_{eff}$$) induces the secondary dressed effect and then the scanning range is divided to double EIT windows, corresponding to no $$E_{F}$$ signal generation shown in Fig. 3(a2) and (a3). Similarly, the three $$E_{F}$$ signal peaks obtained at the intersections of amplitude curve peak with sharp shift dispersion curve. Thirdly, we also observe that the eigenvalues of the dual-dressed states ($$\lambda_{ \pm \pm }$$) determine both the width of the EIT windows and the distance of the lattice states that executes the tunable property as predicted earlier. To sum up, we demonstrate that lattice states can be transformed into self-dressed and dual-dressed type by choosing different x and y coordinates of the standing waves $$G_{3} (x,y)$$ and further modulate $$E_{F}$$ signal intensity distribution which strongly depends on the absorption and dispersion variation of nonlinear coefficient $$\chi^{(3)}$$.

As we have investigated the formation mechanism of $E_{F}$ signal in an ultra-cold atomic ensemble, following this, the property of $E_{F}$ signal at the output surface of the atomic ensemble will be considered. And then the different types EIL of weak light nonlinear $E_{F}$ signal will be realized. The transmission profile is expressed as follows:

2 $$ T(\overrightarrow {{\rho_{{G_{2} + G_{3} (x,y) \pm 0}}^{(3)} }} ) = e^{{ - \chi^{\prime\prime}\nu_{1} L/2c}} e^{{i\chi^{\prime}\nu_{1} L/2c}} $$

**Figure 4:**
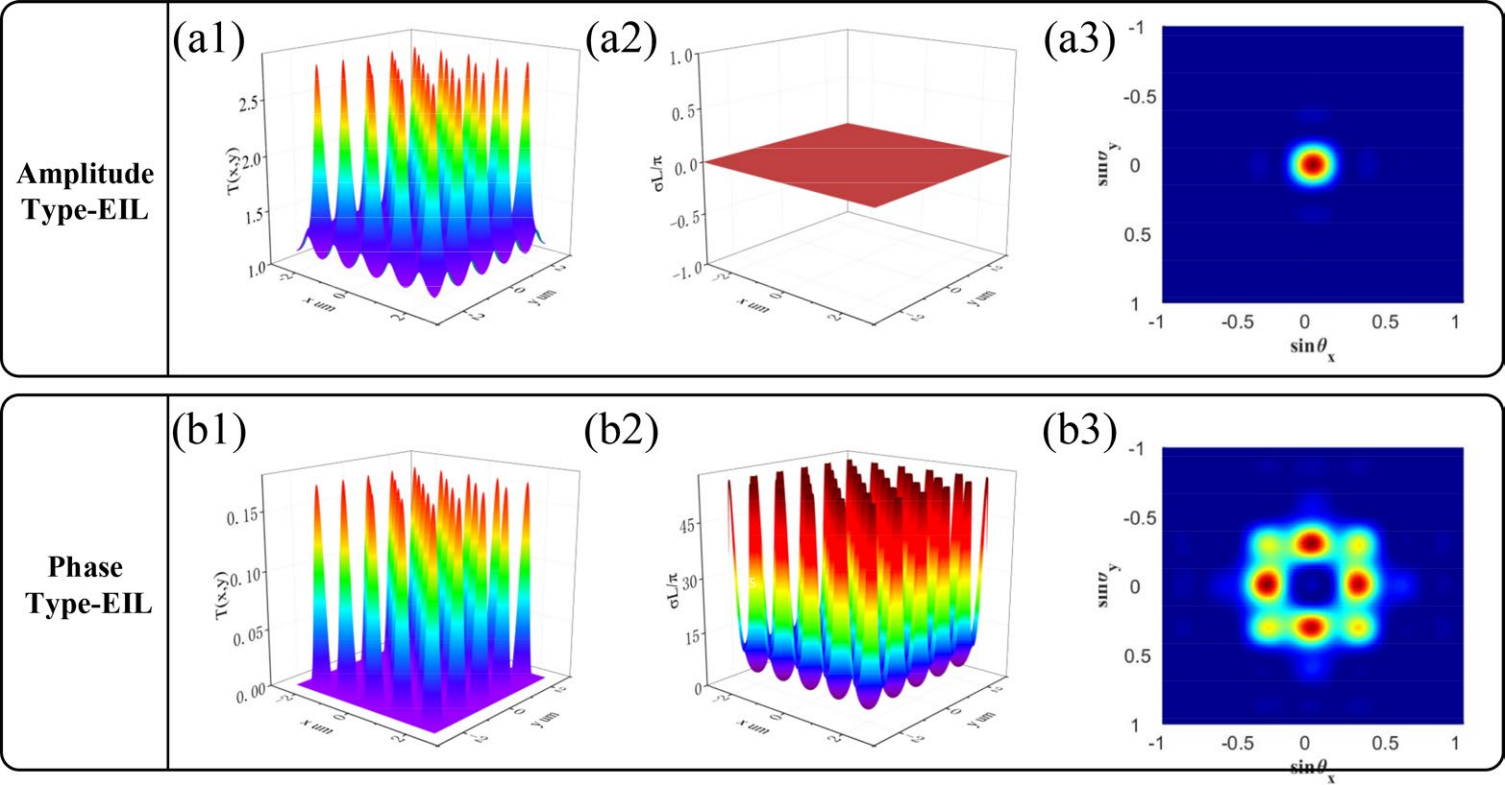
The EIL and far-field diffraction pattern of FWM signal with and without the phase shift. (**a1**) The amplitude EIL with $\Delta_{1} = 0MHz$. (**a2**) The phase distribution of amplitude EIL. (**a3**) The amplitude EIL in far-field region. (**b1**) The hybrid EIL with $\Delta_{1} = 16.95MHz$, $\Delta_{2} = 10MHz$. (**b2**) The phase distribution of hybrid EIL. (**b3**) The phase EIL in far-field region. The other parameters are $G_{1} = 3MHz$, $G_{2} = 10MHz$, $\Omega_{C} = 10MHz$, $\Gamma_{10} = 1MHz$, $\Gamma_{20} = 0.2MHz$, $\Gamma_{30} = 0.2MHz$.

In Eq. (2), L is the propagation distance and the nonlinear coefficient ($\chi = \chi^{\prime} + i\chi^{\prime\prime}$) consists of it’s the imaginary part $\chi^{\prime\prime}$ and the real part $\chi^{\prime}$, which define as the amplitude variation and phase change along the propagation direction. After developing FWM signal EIL at the atomic ensemble surface, we observed that the far-field zero-order diffraction pattern could be effectively transformed into a high-order diffraction pattern subjected to the existence of detuning phase terms. We define $\sigma L = \chi ^{\prime}\nu_{1} L/2c$ as phase shift in order to illuminate the dispersion effect of the phase, which can change the energy distribution. From Fraunhofer’s diffraction theory, the far-field diffraction pattern is proportional to the Fourier transform of T(x, y) (transmission function)^30^. The diffraction field mathematical formulation is given by

3 $$ U(x,y,z) = \frac{{\exp (ikz)\exp [i\frac{k}{2z}(x^{2} + y^{2} )]}}{i\lambda z}\int\limits_{ - \infty }^{\infty } {\int\limits_{ - \infty }^{\infty } {T(\xi ,\eta )\exp [i\frac{k}{2z}(x\xi + y\eta )]d\xi d} } \eta $$

where $(x,y)$ and $(\xi ,\eta )$ are in the diffraction pattern and diffraction aperture planes, respectively. The diffraction intensity distribution is obtained as follows:

4 $$ I(\theta_{x} ,\theta_{Y} ) = \left| {J(\theta_{x} ,\theta_{y} )} \right|^{2} \frac{{\sin^{2} [M\pi a\sin (\theta_{x} /\lambda_{F} )]}}{{M^{2} \sin^{2} [\pi a\sin (\theta_{x} /\lambda_{F} )]}}\frac{{\sin^{2} [N\pi a\sin (\theta_{y} /\lambda_{F} )]}}{{N^{2} \sin^{2} [\pi a\sin (\theta_{y} /\lambda_{F} )]}} $$

where $J(\theta_{x} ,\theta_{y} ) = \iint {dxdyT(x,y)\exp [ - i2\pi xa\sin (\theta_{x} /\lambda_{P} )]\exp [ - i2\pi ya\sin (\theta_{y} /\lambda_{P} )]}$ represents the Fraunhofer diffraction of a single space period, and $\theta_{x}$ ($\theta_{y}$) is the diffraction angle for x (y) direction. M and N are the number of spatial periods along the x and the y-axis of the lattices, which are illustrated by the dressing effect. At first, we set detuning terms at zero ($\Delta_{1} = \Delta_{2} = \Delta_{3} = 0$) and a typical amplitude EIL is realized as shown in Fig. 4(a1). Furthermore, we verify that no phase modulation effect ($\chi^{\prime}{ = 0}$) will attribute to the amplitude EIL shown in Fig. 4(a2). Therefore, we define the EIL of only imaginary part $\chi^{\prime\prime}$ with amplitude effect as amplitude EIL. Moreover, we observed that the transmission of $E_{F}$ is much higher at nodes than at antinodes, which can be explained using dressed theory. At nodes, the intensity of standing wave will reduce to zero and lead to no dressed effect to split level $|1\rangle$. When the probe field resonantly couples with $|0\rangle \to |1\rangle$ at $\Delta_{1} = 0$, the weak light nonlinear $E_{F}$ signal is generated. While the probe field is slightly absorbed at antinodes due to the EIT window introduced by the dressed field, the generation intensity of $E_{F}$ signal is small. As there is no phase shift in the amplitude EIL, the zero-order diffraction intensity is diffracted finitely to high-order as presented in Fig. 4(a3). Secondly, we set $\Delta_{1} = 16.95MHz$, $\Delta_{2} = 10MHz$ in order to compare with $\Delta_{1} = \Delta_{2} = \Delta_{3} = 0$, not only does the imaginary part $\chi^{\prime\prime}$ still carry amplitude effect to the EIL, but also the real part $\chi^{\prime}$ delivers the phase effect. Similarly at antinodes, the low transmission intensity of $E_{F}$ at $\Delta_{{1}} = 16.95MHz$ owes to the scanning position within the EIT window which induced by dual-dressed effects of both $E_{2}$, $E_{2}^{*}$ and standing wave field $G_3 (x,y)$. In addition, the energy of the far-field diffraction pattern is redistributed to first order and four quadrants terms due to the dispersion effect of phase shift function, and the zero-order term reaches the lowest energy in the same time, therefore a far-field phase EIL is realized as shown in Fig. 4(b3). Further, we increase $\Omega_{c}$ to investigate the diffraction effect and found that little intensity of zero-order diffraction terms converted to high-order terms. A consequence of the limited diffraction intensity is that the EIT window width determined by the value of $\Omega_{c}$ cannot be regarded as lattice when $\Omega_{c}$ keep increasing.

## Conclusion

In summary, this study set out to develop a model for an optically tunable EIL of weak light nonlinear FWM signal. The results of this investigation showed that a type conversion is realized from amplitude EIL to synthesized EIL by adding a detuning term. A key strength to form the periodical adjustable lattice states of the present study is that taking advantage of the dressing effects of two-dimensional standing wave. The results of this investigation in the far-field diffraction region shown that the amplitude EIL without the effect of phase shift is precisely close to the ideal sinusoidal amplitude grating. And the dramatic dispersion excursion property that redistributies the diffraction energy from zero-order to higher-order emerged as reliable predictor of the phase EIL. Consequently, the all optically controlled weak light nonlinear signal modulation by lattice states presents EIL and far-field diffraction EIL in a non-destructive process, which highlight the potential usefulness of low medium damage investigation method in the quantum-information field. Further investigation and experimentation into nonlinear EIL are strongly recommended.

## Supplementary information

Supplementary file1 (DOCX 92 kb)
