# Supplementary material for: Nonlinear optical induced lattice in atomic configurations
Source: Sci Rep. 2020 Aug 7;10:13396. doi: 10.1038/s41598-020-67540-2 (PMC7414160; doi:10.1038/s41598-020-67540-2)
Supplement: Supplementary file 1 — Supplementary file1 (DOCX 92 kb) [file 41598_2020_67540_MOESM1_ESM.docx]

**Title: Nonlinear Optical Induced Lattice in Atomic Configurations**

**Author list：**

**Sijia Hui**

Institute of Wide Band Gap Semiconductors, Xi'an Jiaotong University, Xi'an, China

[497077255@qq.com](mailto:497077255@qq.com)

Feng Wen

Institute of Wide Band Gap Semiconductors, Xi'an Jiaotong University, Xi'an, China

Fengwen@xjtu.edu.cn

Xiaojun Yu

School Automation, Northwestern Polytechnical University, Xi’an, 710072, China

XJYU@nwpu.edu.cn

Zhiping Dai

College of Physics and Electronic Engineering, Hengyang Normal University, Hengyang, 421002 China

[hydzp@hynu.edu.cn](mailto:hydzp@hynu.edu.cn)

Irfan Ahmed

Department of Electrical Engineering, Sukkur IBA University, Sukkur, 65200, Pakistan

[iahmed8-c@my.cityu.edu.cn](mailto:iahmed8-c@my.cityu.edu.cn)

Yunpeng Su

State-owned Sida Machinery Manufacturing, [712201](http://www.youbian.com/712201/), Xianyang, China

[1013720982@qq.com](mailto:1013720982@qq.com)

Yanpeng Zhang

Institute of Wide Band Gap Semiconductors, Xi'an Jiaotong University, Xi'an, China

ypzhang@mail.xjtu.edu.cn

Hongxing Wang

Institute of Wide Band Gap Semiconductors, Xi'an Jiaotong University, Xi'an, China

hxwang@mail.xjtu.edu.cn

**Method**

**Generation of the FWM signal in the Atomic Ensemble.** In the interaction picture, the effective Hamiltonian under the electric-dipole approximation and the rotating-wave approximation is expressed as ():

$H=G_{1}e^{-i\Delta_{1}t}|0\rangle\langle1|+G_{2}e^{-i\Delta_{2}t}|1\rangle\langle2|+G_{2}^{*}e^{-i\Delta_{2}t}|1\rangle\langle2|+G_{3}e^{-i\Delta_{3}t}|1\rangle\langle3|+h.c.$ (1)

Where are the Rabi frequencies of the optical pumping field, and the laser-field detuning defined as $\Delta_{1}=\omega_{1}-\omega_{10}$, $\Delta_{2}=\omega_{2}-\omega_{21}$, and $\Delta_{3}=\omega_{2}-\omega_{31}$ from the transitions $|0\rangle\to|1\rangle$, $|1\rangle\to|2\rangle$, $|1\rangle\to|3\rangle$ with $\omega_{ij}=\omega_{i}-\omega_{j}$, $(i,j=0,1,2,3)$.

By using the Liouville equation, the coupled system equations are obtained:

$\rho^{.} _{01}=(\gamma_{10}-i\Delta_{1})\rho_{01}-iG_{1}(\rho_{00}-\rho_{11})+\frac{i}{2}(G_{2}+G_{2}^{*})\rho_{02}+iG_{3}(x,y)\rho_{03}$ (2)

$\rho^{.} _{02}=(\gamma_{20}-i\Delta_{12})\rho_{02}+\frac{i}{2}(G_{2}+G_{2}^{*})\rho_{01}-iG_{1}\rho_{12}$ (3)

$\rho^{.} _{03}=(\gamma_{30}-i\Delta_{13})\rho_{03}+iG_{3}\rho_{01}-iG_{1}\rho_{13}$ (4)

$\rho^{.} _{12}=(\gamma_{21}-i\Delta_{2})\rho_{12}-\frac{i}{2}(G_{2}+G_{2}^{*})(\rho_{22}-\rho_{11})-iG_{1}\rho_{02}-iG_{3}(x,y)\rho_{13}$ (5)

$\rho^{.} _{13}=(\gamma_{31}-i\Delta_{3})\rho_{13}-iG_{3}(x,y)(\rho_{33}-\rho_{11})-\frac{i}{2}(G_{2}+G_{2}^{*})\rho_{13}-iG_{1}\rho_{03}$ (6)

$\rho^{.} _{23}=(\gamma_{32}-i\Delta_{23})\rho_{23}-\frac{i}{2}(G_{2}+G_{2}^{*})\rho_{13}+iG_{3}(x,y)\rho_{12}$ (7)

where $\sum_{i=0}^{3} \rho_{ii}=1$, $\rho_{ij}=\rho_{ji}^{*}$ and $\Delta_{12}=\Delta_{1}+\Delta_{2}$, $\Delta_{13}=\Delta_{1}+\Delta_{3}$ and $\Delta_{23}=\Delta_{2}-\Delta_{3}$. $\gamma_{20}$, $\gamma_{30}$, $\gamma_{32}$ are the decoherence rates and $\gamma_{10}$, $\gamma_{21}$, $\gamma_{31}$ are the decay rates of upper levels.

By solving Eqs (2–7), with the assumption that the atomic ensemble is initially in its ground state, the dressed density matrix at $\omega_{F}$ is obtained:

$\rho_{G_{2}+G_{3}(x,y)\pm0}^{(3)}=\frac{G_{a}}{d_{2}(d_{1}+\left|G_{2}\right|^{2}/d_{2}+\left|G_{3}(x,y)\right|^{2}/d_{3})^{2}}$ (8)

where $G_{a}=-iG_{1}G_{2}(G_{2}^{'})^{*}\exp(ik_{F}\cdot r)$, $G_{1}$, $G_{2}$, and $G_{2}^{*}$ are the Rabi frequencies of $E_{1}$, $E_{2}$, $E_{2}^{*}$, respectively, and $d_{1}=\Gamma_{10}+i\Delta_{1}$ $d_{2}=\Gamma_{20}+i(\Delta_{1}+\Delta_{2})$, $d_{3}=\Gamma_{30}+i(\Delta_{1}+\Delta_{3})$. $\Gamma_{ij}$ is the decay rate between the energy level $|i\rangle$ and $|j\rangle$ $i,j=0,1,2,3$. The expression of Rabi frequency of standing waves is $\left|G_{3}(x,y)\right|^{2}=\left|\Omega_{c}\sin(\pi x/a)\right|^{2}+\left|\Omega_{c}\sin(\pi y/a)\right|^{2}$, where $\Omega_{c}$ is the amplitudes of the two laser fields along the X and Y direction and is assumed to be real for simplicity. a (b) is the corresponding spatial period, as which can be made arbitrarily smaller or larger by varying the angle between the two wave vectors of $E_{3}(x1)$ and $E_{3}(x2)$ ($E_{3}^{*}(y1)$ and $E_{3}^{*}(y2)$).
